# Variation in Cancer Incidence Rates Among Non-Hispanic Black Individuals Disaggregated by Nativity and Birthplace, 2005-2017: A Population-Based Cancer Registry Analysis

**DOI:** 10.3389/fonc.2022.857548

**Published:** 2022-04-08

**Authors:** Adana A. M. Llanos, Jie Li, Jennifer Tsui, Joseph Gibbons, Karen Pawlish, Fechi Nwodili, Shannon Lynch, Camille Ragin, Antoinette M. Stroup

**Affiliations:** ^1^ Department of Epidemiology, Mailman School of Public Health, Columbia University Irving Medical Center, New York, NY, United States; ^2^ Cancer Population Science, Herbert Irving Comprehensive Cancer Center, New York, NY, United States; ^3^ New Jersey State Cancer Registry, New Jersey Department of Health, Trenton, NJ, United States; ^4^ Department of Population and Public Health, Keck School of Medicine, University of Southern California, Los Angeles, CA, United States; ^5^ Department of Sociology, San Diego State University, San Diego, CA, United States; ^6^ Rutgers University School of Arts and Sciences, Douglass Residential College, New Brunswick, NJ, United States; ^7^ Cancer Prevention and Control Program, Fox Chase Cancer Center-Temple Health, Philadelphia, PA, United States; ^8^ Department of Biostatistics and Epidemiology, Rutgers School of Public Health, Piscataway, NJ, United States; ^9^ Cancer Prevention and Control, Rutgers Cancer Institute of New Jersey, New Brunswick, NJ, United States

**Keywords:** cancer surveillance, cancer incidence, non-Hispanic Black subgroups, within-group differences, cancer inequities, population-based study, cancer registry data

## Abstract

**Objectives:**

Compared to other racial and ethnic groups, little to no disaggregated cancer incidence data exist for subgroups of non-Hispanic Blacks (NHBs), despite heterogeneity in sociodemographic characteristics and cancer risk factors within this group. Our objective was to examine age-adjusted cancer incidence by nativity and birthplace among NHB cancer cases diagnosed in New Jersey.

**Methods:**

Race, ethnicity, and birthplace data from the New Jersey State Cancer Registry were used to classify NHB cancer cases diagnosed between 2005-2017. Thirteen waves of population estimates (by county, nativity, gender, age-group) were derived from the American Community Survey using Integrated Public-Use Microdata to approximate yearly demographics. Age-adjusted cancer incidence rates (overall and by site) by birthplace were generated using SEER*Stat 8.3.8. Bivariate associations were assessed using chi-square and Fisher’s exact tests. Trend analyses were performed using Joinpoint 4.7.

**Results:**

Birthplace was available for 62.3% of the 71,019 NHB cancer cases. Immigrants represented 12.3%, with African-born, Haitian-born, Jamaican-born, ‘other-Caribbean-born’, and ‘other-non-American-born’ accounting for 18.5%, 17.7%, 16.5%, 10.6%, and 36.8%, respectively. Overall, age-adjusted cancer incidence rates were lower for NHB immigrants for all sites combined and for several of the top five cancers, relative to American-born NHBs. Age-adjusted cancer incidence was lower among immigrant than American-born males (271.6 vs. 406.8 per 100,000) and females (191.9 vs. 299.2 per 100,000). Age-adjusted cancer incidence was lower for Jamaican-born (114.6 per 100,000) and other-Caribbean-born females (128.8 per 100,000) than African-born (139.4 per 100,000) and Haitian-born females (149.9 per 100,000). No significant differences in age-adjusted cancer incidence were observed by birthplace among NHB males. Age-adjusted cancer incidence decreased for all sites combined from 2005-2017 among American-born males, immigrant males, and American-born females, while NHB immigrant female rates remained relatively stable.

**Conclusions:**

There is variation in age-adjusted cancer incidence rates across NHB subgroups, highlighting the need for more complete birthplace information in population-based registries to facilitate generating disaggregated cancer surveillance statistics by birthplace. This study fills a knowledge gap of critical importance for understanding and ultimately addressing cancer inequities.

## Introduction

Non-Hispanic Blacks (NHBs) represent the second-largest racial/ethnic minority group in the United States (US)—comprising approximately 13.4% of the population, as of 2019 (1). The NHB population is a diverse group that includes descendants of enslaved Africans brought to the Americas during the transatlantic slave trade beginning in the 16^th^ century and immigrants arriving more recently from across the African diaspora and their descendants. Immigrants account for 10% of the NHB population, with Jamaican-born, Haitian-born, and Nigerian-born individuals accounting for the three largest NHB subgroups by birthplace (2, 3). Inequities in cancer incidence, mortality, and survival exist by race and ethnicity for many cancer sites (4), with evidence showing the highest sex-specific cancer incidence among NHB males and the highest sex-specific mortality among NHB females (5). Furthermore, 5-year relative survival for all cancers combined is lowest among NHBs (5, 6). Despite the knowledge that NHBs disproportionately shoulder the burden of cancer (7) and that NHBs in the US are not a monolithic group (2), little to no cancer surveillance statistics exist for subgroups of NHBs, in contrast to subgroups of Asian American/Pacific Islander (8–15) and Hispanic/Latinx individuals (13, 15–17).

While limited data are currently available on cancer incidence among disaggregated NHB groups (18, 19), a handful of studies show significant heterogeneity in cancer mortality by NHB subgroup in the US (20–23). African-born NHBs have higher incidence of infection-related cancers (18) and Caribbean-born NHBs have lower risk of cancer mortality (20–23) compared to American-born NHBs, suggesting that the aggregation of all NHBs into a singular group in cancer surveillance masks within-group differences and limits the ability to inform targeted intervention needs for higher-risk communities.

Cancer surveillance programs in the US have successfully begun generating cancer profiles for subgroups of Asian American/Pacific Islander (24) and Hispanic/Latinx (25), yet, to our knowledge, no such profiles exist for NHB subgroups. As a starting point, we examined variation in age-adjusted cancer incidence by birthplace among NHB cancer cases diagnosed in New Jersey (NJ)–the fourth most racially/ethnically diverse state in the US, with substantial socioeconomic, geographic, and subgroup diversity within the NHB population. Further, we highlight methodologic limitations related to generation of race and ethnicity subgroup data and next steps for standardization and systematic data collection of NHB subgroups.

## Methods

Age-adjusted cancer incidence rates (overall and by site) were generated using cancer incidence data from the New Jersey State Cancer Registry (NJSCR). The NJSCR is a population-based registry that collects data on all cancer cases diagnosed in NJ. NJSCR consistently receives awards for data quality and completeness from the North American Association of Central Cancer Registries (NAACCR), the Centers for Disease Control and Prevention (CDC) National Program of Cancer Registries (NPCR), and the National Cancer Institute’s (NCI) Surveillance, Epidemiology, and End Results Program (SEER). The Rutgers University Institutional Review Board approved this study.

All NHB cancer cases diagnosed from 2005-2017 were included. Subgroup categories (American-born, NHB immigrants, and unknown) were created using country of birth and US birth state data from NJSCR. Individuals born in the US and its territories were considered American-born (26); otherwise, they were grouped as NHB immigrants or unknown. NHB immigrant subgroups by birthplace (African-born, Haitian-born, Jamaican-born, other Caribbean-born, and other NHB immigrants) were created using nativity and country of birth. The group classified as ‘other Caribbean-born’ included individuals born in Caribbean countries other than Haiti and Jamaica (e.g., Trinidad and Tobago, Grenada, Antigua and Barbuda, Barbados), while other NHB immigrants included individuals not classified in one of the previous subgroups. For NHB population estimates, we used Integrated Public-Use Microdata (IPUMS) from the American Community Survey (ACS, 1-year waves) for 2005–2017. To estimate the NHB population: 1) we considered individuals classified as NHB in some way (e.g., multiracial–NHB and another race) as exclusively NHB in this study (3), and 2) we classified major origin sites of NHB immigrants residing in NJ, including Haitian, Jamaican, other Caribbean countries, and sub-Saharan African.

Cancer incidence data and population estimates were processed in SEER*Prep 2.5.7 to create SEER*Stat databases. We used chi-square and Fisher’s exact tests to examine associations between NHB subgroup and age group (0-39, 40-64, or ≥65 years), gender (male or female), vital status (alive or deceased), cancer site (SEER Site Recode based on International Classification of Diseases for Oncology, 3rd edition), cancer stage (SEER summary stage), county of residence (health care regions), and census tract poverty (<5%, 5-<10%, 10-<20%, or ≥20%). Bivariate analyses were completed in SAS 9.4. Statistical significance was set at p<0.05.

Incidence rates for all cancers combined and rates by cancer site across NHB subgroups and gender were computed in SEER*Stat 8.3.8. Rates are per 100,000 and age-adjusted to the 2000 US standard population. Trend analysis to examine estimated annual percent change in age-adjusted incidence rates over the study period was performed using Joinpoint 4.7. We used the log-linear model and Monte Carlo Permutation method for significance tests, and the significance level was set at *P*<0.05.

## Results

### Descriptive Statistics of NHB Cancer Cases Diagnosed in NJ

**Figure 1:**
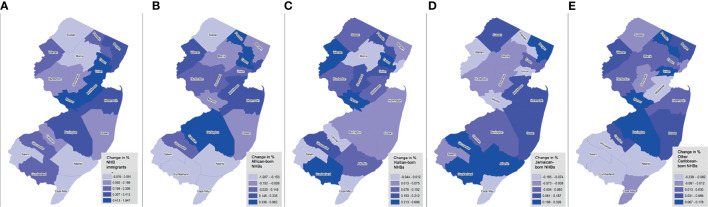
Trends in NHB subgroup populations by birthplace across New Jersey counties, 2005-2017. Shown as change in percent of foreign-born NHBs (overall) **(A)**, African-born **(B)**, Haitian-born **(C)**, Jamaican-born **(D)**, and other Caribbean-born **(E)**, by county.

Between January 1, 2005 and December 31, 2017, there were 71,019 incident cancers diagnosed among NHB individuals in NJ (**Table 1**). Among those with documented birthplace, NHB immigrants represented 12.3%, while American-born represented 87.7%. Among NHB immigrants, 18.5% were born in an African country, 17.7% in Haiti, 16.5% in Jamaica, 10.6% in another Caribbean country (not Haiti or Jamaica), and 36.8% elsewhere (not in the US or an African or Caribbean country). NHB cases with unknown birthplace (37.7%) were younger, diagnosed at earlier stages, and alive at the end of follow-up. The proportion of cases with unknown birthplace varied across the study period, ranging from as low as 28.6% (in 2005) to as high as 50.2% (in 2017) (**Supplementary Table 1**). Compared to American-born NHBs, significantly larger proportions of cases among NHB immigrants were diagnosed at 0-39 years (6.1% vs. 4.1%) and 40-64 years (50.3% vs. 40.9%), and a smaller proportion at age ≥65 years (43.6% vs. 55.0%). Stage distribution was similar between American-born and NHB immigrant cases. There was some variation by birthplace in terms of regions of New Jersey where the largest proportions of NHB cases resided. Larger proportions of NHB immigrant groups regions generally resided in Essex and Passaic counties, Bergen and Hudson counties, and Middlesex and Union counties. NHB immigrant populations increased most in a few contiguous counties, from Essex to Mercer County (**Figure 1**). However, the change in representation of specific ancestries of NHB immigrants varies across the state. For example, southern counties experienced an increase in Haitian and Jamaican populations during the study period.

**Table 1:** Descriptive statistics of non-Hispanic Black (NHB) cancer cases diagnosed in New Jersey by nativity and birthplace, 2005-2017.

Sociodemographic characteristic	All NHB cancer cases^1^	American-born^2^	NHB immigrants^3-7^					Unknown birthplace^8^	P ^(2,3,4,5,6,7,8)^
			African-born^3^	Haitian-born^4^	Jamaican-born^5^	Other Caribbean-born^6^	Other immigrants^7^		
	N (%)	n (%)	n (%)	n (%)	n (%)	n (%)	n (%)	n (%)	
Total	71019 (100.0)	38834 (54.7)	1004 (18.5)	960 (17.7)	896 (16.5)	574 (10.6)	1999 (36.8)	26752 (37.7)	
Age Group									<0.0001*
0-39	3772 (5.3)	1600 (4.1)	110 (11.0)	73 (7.6)	46 (5.1)	34 (5.9)	69 (3.5)	1840 (6.9)	
40-64	33540 (47.2)	15886 (40.9)	613 (61.1)	475 (49.5)	459 (51.2)	324 (56.4)	862 (43.1)	14921 (55.8)	
≥65	33707 (47.5)	21348 (55.0)	281 (28.0)	412 (42.9)	391 (43.6)	216 (37.6)	1068 (53.4)	9991 (37.3)	
Gender									<0.0001*
Male	35522 (50.0)	18687 (48.1)	566 (56.4)	457 (47.6)	425 (47.4)	272 (47.4)	989 (49.5)	14126 (52.8)	
Female	35497 (50.0)	20147 (51.9)	438 (43.6)	503 (52.4)	471 (52.6)	302 (52.6)	1010 (50.5)	12626 (47.2)	
Primary Site^¤^									
*Male*									<0.0001*^
Prostate	14034 (39.5)	4677 (25.0)	239 (42.2)	188 (41.1)	164 (38.6)	125 (46.0)	324 (32.8)	8317 (58.9)	
Lung and Bronchus	4271 (12.0)	3493 (18.7)	30 (5.3)	32 (7.0)	43 (10.1)	14 (5.1)	127 (12.8)	532 (3.8)	
Colon and Rectum	3363 (9.5)	2000 (10.7)	62 (11.0)	45 (9.8)	43 (10.1)	33 (12.1)	112 (11.3)	1068 (7.6)	
Kidney and Renal Pelvis	1510 (4.3)	760 (4.1)	18 (3.2)	6 (1.3)	14 (3.3)	15 (5.5)	25 (2.5)	672 (4.8)	
Urinary Bladder	1161 (3.3)	707 (3.8)	6 (1.1)	4 (0.9)	15 (3.5)	10 (3.7)	27 (2.7)	392 (2.8)	
Other	11183 (31.5)	7050 (37.7)	211 (37.3)	182 (39.8)	146 (34.4)	75 (27.6)	374 (37.8)	3145 (22.3)	
*Female*									<0.0001*
Breast	10864 (30.6)	5130 (25.5)	145 (33.1)	168 (33.4)	150 (31.8)	120 (39.7)	251 (24.9)	4900 (38.8)	
Lung and Bronchus	4119 (11.6)	3320 (16.5)	21 (4.8)	25 (5.0)	24 (5.1)	9 (3.0)	84 (8.3)	636 (5.0)	
Colon and Rectum	3710 (10.5)	2090 (10.4)	29 (6.6)	36 (7.2)	47 (10.0)	31 (10.3)	117 (11.6)	1360 (10.8)	
Corpus and Uterus NOS	2452 (6.9)	1252 (6.2)	34 (7.8)	45 (8.9)	35 (7.4)	19 (6.3)	104 (10.3)	963 (7.6)	
Pancreas	1200 (3.4)	963 (4.8)	24 (5.5)	24 (4.8)	17 (3.6)	5 (1.7)	41 (4.1)	126 (1.0)	
Other	13152 (37.1)	7392 (36.7)	185 (42.2)	205 (40.8)	198 (42.0)	118 (39.1)	413 (40.9)	4641 (36.8)	
SEER Summary Stage									<0.0001*
In Situ	4904 (6.3)	1771 (4.3)	41 (3.7)	47 (4.4)	62 (6.4)	33 (5.3)	57 (2.7)	2893 (9.3)	
Local	30060 (38.5)	12148 (29.5)	387 (35.3)	346 (32.6)	365 (37.4)	218 (35.2)	576 (27.7)	16020 (51.6)	
Regional	14209 (18.2)	8145 (19.8)	207 (18.9)	223 (21.0)	182 (18.7)	146 (23.6)	348 (16.7)	4958 (16.0)	
Distant	18515 (23.7)	13278 (32.3)	303 (27.7)	294 (27.7)	261 (26.8)	153 (24.7)	766 (36.8)	3460 (11.1)	
Unknown/Unstaged	10319 (13.2)	5792 (14.1)	157 (14.3)	150 (14.2)	105 (10.8)	69 (11.1)	332 (16.0)	3714 (12.0)	
Health Care Region (counties)									<0.0001*^
Region 1 (Sussex, Warren)	334 (0.5)	169 (0.4)	9 (0.9)	5 (0.5)	8 (0.9)	3 (0.5)	11 (0.6)	129 (0.5)	
Region 2 (Essex, Passaic)	21017 (29.6)	11614 (29.9)	318 (31.7)	377 (39.3)	325 (36.3)	188 (32.8)	861 (43.1)	7334 (27.4)	
Region 3 (Bergen, Hudson)	7139 (10.1)	4212 (10.8)	123 (12.3)	102 (10.6)	142 (15.8)	140 (24.4)	240 (12.0)	2180 (8.1)	
Region 4 (Morris, Somerset)	2704 (3.8)	1319 (3.4)	78 (7.8)	28 (2.9)	83 (9.3)	41 (7.1)	89 (4.5)	1066 (4.0)	
Region 5 (Hunterdon, Mercer)	5123 (7.2)	2622 (6.8)	91 (9.1)	70 (7.3)	46 (5.1)	8 (1.4)	108 (5.4)	2178 (8.1)	
Region 6 (Middlesex, Union)	11174 (15.7)	6203 (16.0)	269 (26.8)	295 (30.7)	183 (20.4)	140 (24.4)	315 (15.8)	3769 (14.1)	
Region 7 (Monmouth, Ocean)	4247 (6.0)	2256 (5.8)	33 (3.3)	38 (4.0)	38 (4.2)	27 (4.7)	134 (6.7)	1721 (6.4)	
Region 8 (Burlington, Camden)	11687 (16.5)	6222 (16.0)	58 (5.8)	24 (2.5)	41 (4.6)	19 (3.3)	154 (7.7)	5169 (19.3)	
Regions 9 and 10 (Atlantic, Cape May, Cumberland, Gloucester, and Salem)	7560 (10.6)	4204 (10.8)	25 (2.5)	21 (2.2)	30 (3.3)	8 (1.4)	87 (4.4)	3185 (11.9)	
Unknown	34 (0.1)	13 (0.03)	0 (0.0)	0 (0.0)	0 (0.0)	0 (0.0)	0 (0.0)	21 (0.1)	
Census Tract Poverty									<0.0001*^
<5%	11567 (16.3)	5616 (14.5)	200 (19.9)	154 (16.0)	173 (19.3)	130 (22.6)	368 (18.4)	4926 (18.4)	
5 - <10%	13781 (19.4)	6982 (18.0)	250 (24.9)	177 (18.4)	187 (20.9)	122 (21.3)	346 (17.3)	5717 (21.4)	
10 - <20%	20580 (29.0)	10989 (28.3)	298 (29.7)	338 (35.2)	296 (33.0)	181 (31.5)	651 (32.6)	7827 (29.3)	
≥20%	24969 (35.2)	15183 (39.1)	256 (25.5)	291 (30.3)	239 (26.7)	141 (24.6)	629 (31.5)	8230 (30.8)	
Unknown	122 (0.2)	64 (0.2)	0 (0.0)	0 (0.0)	1 (0.1)	0 (0.0)	5 (0.3)	52 (0.2)	
Vital Status (as of December 2017)									<0.0001*
Alive	33869 (47.7)	7755 (20.0)	646 (64.3)	484 (50.4)	480 (53.6)	351 (61.1)	254 (12.7)	23899 (89.3)	
Deceased	37150 (52.3)	31079 (80.0)	358 (35.7)	476 (49.6)	416 (46.4)	223 (38.9)	1745 (87.3)	2853 (10.7)	

P-values generated using bivariate analysis comparing sociodemographic characteristics by birthplace and nativity.

^1^Based on NAACCR NHIA, NJSCR Race1, and Birthplace (state and county): NHIA = 0 (Non-Hispanic) and Race1 = 2 (Black).

^2^American-Born: NHB cancer cases born in the United States of America and its territories.

^3^African-born: NHB cancer cases born in any nation of Africa.

^4^Haitian-born: NHB cancer cases born in Haiti.

^5^Jamaican-born: NHB cancer cases born in Jamaica.

^6^Other Caribbean-born: NHB cancer cases born in any Caribbean nation other than Haiti and Jamaica.

^7^Other non-US-born: NHB cancer cases not included in the groups above (non-US-born).

^8^Unknown birthplace: NHB cancer cases missing data on birthplace.

*P-values significant with and without inclusion of cases with unknown birthplace.

^P-values generated using Chi-square and Fisher’s exact tests.

^¤^Top 5 cancer sites based on NHB age-adjusted (6 age groups) invasive cancer incidence rates 2005-2017 by gender.

As of December 31, 2017, 52.3% of NHB cancer cases overall were deceased and there was an indication that the proportion of American-born cases who died was larger than that of NHB immigrants (80.0% vs. 59.2%). Only 10.7% of deceased cases were missing birthplace information. We also found that slightly higher proportions of NHB immigrants were lost to follow-up in earlier years of the study period than American-born cases, but follow-up rates were similar in more recent years (**Supplementary Table 2**).

### Age-Adjusted Cancer Incidence Among NHBs in NJ

Relative to American-born individuals, NHB immigrants had lower cancer incidence rates for all cancer sites combined and for several of the top five cancers diagnosed among NHBs. Among NHB males, the age-adjusted cancer incidence rate for all sites combined was 607.9/100,000 (**Table 2**). Age-adjusted cancer incidence was higher in American-born NHB males than immigrant NHB males (271.6 vs. 406.8/100,000). Notably, cancer incidence for all sites combined among other NHB immigrant males was exceptionally high (803.4/100,000). While prostate cancer incidence did not differ among American-born and immigrant NHB males, incidence rates for several cancer sites were lower among NHB immigrants than among American-born males: lung and bronchus (26.2 vs. 77.5/100,000), colon and rectum (30.3 vs. 44.9/100,000), kidney and renal pelvis (7.9 vs. 15.8/100,000), bladder (7.7 vs. 17.1/100,000), and other sites (95.8 vs. 149.7/100,000). Although none of the differences reached statistical significance, cancer incidence rates varied among NHB males across immigrant subgroups.

**Table 2:** Age-adjusted cancer incidence (per 100,000) among non-Hispanic Black (NHB)^1^ individuals in New Jersey by nativity and birthplace, and by gender, 2005-2017.

Gender / Cancer Site^¤^	All NHB^1^	American-born^2^	NHB immigrants^3-7^	African-born^3^	Haitian-born^4^	Jamaican-born^5^	Other Caribbean-born^6^	Other NHB immigrants^7^
	Rate (95% CI)	Rate (95% CI)	Rate (95% CI)	Rate (95% CI)	Rate (95% CI)	Rate (95% CI)	Rate (95% CI)	Rate (95% CI)
Male								
All sites combined	607.9 (601.3, 614.5)	406.8 (400.8, 412.9)	271.6 (259.8, 283.9)*	197.2 (176.1, 220.1)	195.2 (174.7, 217.5)	175.8 (156.8, 196.7)	175.1 (151.7, 202.1)	803.4 (750.3, 859.3)^††^
Prostate	235.9 (231.9, 240.0)	101.9 (98.9, 104.9)	103.8 (96.6, 111.3)	84.2 (70.8, 99.4)	83.6 (70.1, 99.1)	64.0 (53.4, 76.3)	80.4 (65.7, 98.6)	271.2 (240.6, 304.7)
Lung and bronchus	77.5 (75.1, 79.9)	77.5 (74.8, 80.1)	26.2 (22.6, 30.2)*	9.6 (6.0, 15.1)	15.4 (9.9, 23.0)	20.1 (13.9, 28.5)	8.9 (4.5, 18.0)	101.3 (83.2, 122.2)^††^
Colon and rectum	59.4 (57.3, 61.6)	44.9 (42.9, 46.9)	30.3 (26.3, 34.6)*	22.6 (15.6, 31.6)	19.6 (13.3, 27.8)	17.2 (11.8, 24.5)	20.9 (13.6, 32.4)	92.7 (75.2, 113.2)^††^
Kidney and renal pelvis	24.4 (23.2, 25.8)	15.8 (14.7, 17.0)	7.9 (6.0, 10.3)*	8.4 (4.1, 15.1)	2.3 (0.8, 5.7)	5.0 (2.7, 9.5)	12.4 (4.2, 27.2)	19.7 (12.2, 30.1)
Urinary bladder	22.5 (21.2, 23.9)	17.1 (15.9, 18.5)	7.7 (5.6, 10.2)*	4.5 (1.2, 10.7)	2.8 (0.6, 7.5)	5.5 (2.8, 10.3)	6.9 (3.0, 15.7)	25.6 (16.4, 38.0)
Other	188.2 (184.5, 191.8)	149.7 (146.1, 153.3)	95.8 (88.9, 103.2)*	68.0 (56.1, 81.7)	71.6 (60.0, 84.9)	64.1 (52.0, 78.3)	45.6 (34.9, 59.8)	292.9 (261.4, 327.1)^††^
Female								
All sites combined	431.1 (426.6, 435.6)	299.2 (295.1, 303.4)	191.9 (184.3, 199.9)*	139.4 (124.9, 155.1)	149.9 (136.3, 164.7)^†^	114.6 (103.7, 126.7)^†^	128.8 (111.2, 148.9)^†^	614.9 (575.1, 656.8)^††^
Breast	130.1 (127.6, 132.6)	76.2 (74.1, 78.3)	52.4 (48.8, 56.4)*	40.5 (33.6, 48.6)^†^	44.7 (38.0, 52.5)	33.8 (28.4, 40.4)^†^	45.7 (37.5, 56.1)	137.9 (120.3, 157.6)
Lung and bronchus	50.4 (48.8, 51.9)	49.0 (47.4, 50.7)	12.4 (10.5, 14.6)*	8.0 (4.5, 12.9)	8.6 (5.4, 13.2)	5.6 (3.5, 9.3)^†^	3.7 (1.6, 9.2)^†^	53.8 (42.4, 67.5)
Colon and rectum	45.6 (44.1, 47.1)	31.2 (29.9, 32.6)	18.5 (16.2, 21.1)*	8.6 (5.4, 13.0)^†^	10.6 (7.3, 15.1)^†^	11.4 (8.2, 15.9)^†^	11.7 (7.8, 18.4)	73.1 (59.8, 88.6)^††^
Corpus and uterus NOS	29.2 (28.0, 30.4)	18.3 (17.3, 19.3)	16.6 (14.4, 19.0)	13.2 (8.8, 19.0)	13.0 (9.4, 17.8)	8.4 (5.8, 12.4)^†^	7.8 (4.6, 14.0)^†^	61.2 (49.2, 75.3)^††^
Pancreas	15.0 (14.1, 15.9)	14.4 (13.5, 15.3)	8.6 (7.0, 10.5)*	8.4 (4.9, 13.4)	8.6 (5.4, 13.2)	4.3 (2.4, 7.7)	1.6 (0.5, 6.6)^†^	28.5 (20.1, 39.2)^††^
Other	160.9 (158.1, 163.7)	110.1 (107.6, 112.6)	83.5 (78.2, 89.0)*	60.7 (51.1, 71.6)^†^	64.5 (55.3, 74.9)^†^	51.2 (43.5, 60.1)^†^	58.3 (44.5, 75.3)^†^	260.5 (234.3, 288.9)^††^

^1^Based on NAACCR NHIA, NJSCR Race1, and Birthplace (state and county): NHIA = 0 (Non-Hispanic) and Race1 = 2 (Black).

^2^American-Born: NHB cancer cases born in the United States of America and its territories.

^3^African-born: NHB cancer cases born in any nation of Africa.

^4^Haitian-born: NHB cancer cases born in Haiti.

^5^Jamaican-born: NHB cancer cases born in Jamaica.

^6^Other Caribbean-born: NHB cancer cases born in any Caribbean nation other than Haiti and Jamaica.

^7^Other non-US-born: NHB cancer cases not included in the groups above (non-US-born).

^¤^Top 5 cancer sites based on NHB age-adjusted (6 age groups) invasive cancer incidence rates 2005-2017 by gender.

*Indicates statistically significant difference in incidence rates in comparison to American-born NHBs.

^^†^^Incidence rates in these immigrant subgroups were significantly lower than rates for NHB immigrants combined.

^††^Incidence rates for these cancers were significantly higher among individuals in the Other NHB immigrant subgroup compared to rates for all NHBs combined.

Among NHB females, age-adjusted cancer incidence for all sites combined was 431.1/100,000. Age-adjusted cancer incidence was lower among immigrant NHB females than American-born females (191.9 vs. 299.2/100,000). Similar to males, cancer incidence for all sites combined among other NHB immigrant females was quite high (614.9/100,000). Cancer incidence rates among NHB females varied across immigrant subgroups: incidence was lower among Jamaican-born (114.6/100,000) and other Caribbean-born females (128.8/100,000) compared to African-born (139.4/100,000) and Haitian-born females (149.9/100,000). All site-specific cancer incidence rates (except corpus and uterine) were lower among immigrant than American-born NHB females: breast (52.4 vs. 76.2/100,000), lung and bronchus (12.4 vs. 49.0/100,000), colon and rectum (18.5 vs. 31.2/100,000), pancreas (8.6 vs. 14.4/100,000), and other sites (83.5 vs. 110.1/100,000).

### Age-Adjusted Cancer Incidence Trends Among NHBs in NJ

We observed significant decreasing trends in age-adjusted cancer incidence for all sites combined from 2005-2017 among American-born males, immigrant males, and American-born females, while NHB immigrant female rates remained relatively stable (**Table 3**). Among American-born NHB males, age-adjusted cancer incidence for all sites combined decreased by 4.57% per year. This group also experienced statistically significant reductions in age-adjusted incidence for several site-specific cancers during this time: prostate (-9.37%), lung and bronchus (-3.70%), colon and rectum (-4.85%). Although not statistically significant, age-adjusted kidney and renal pelvis cancer incidence decreased by 2.15% per year from 2005-2015 and by 26.22% from 2015-2017. Overall, among NHB immigrant males, age-adjusted cancer incidence for all sites combined decreased by 4.76% per year, and prostate cancer by 9.13% per year. A trend towards decreasing cancer incidence among African-born, Jamaican-born, and other Caribbean-born males was observed, but no estimates reached statistical significance. Conversely, Haitian-born and other NHB immigrant males experienced large reductions in prostate cancer incidence from 2005-2017 (15.16% and 24.27%, respectively).

**Table 3:** Age-adjusted cancer incidence trends for top 5 cancer sites among non-Hispanic Black (NHB)^1^ individuals in New Jersey by nativity and birthplace, and by gender, 2005-2017.

Gender / Cancer Site^¤^	American-born^2^		NHB immigrants		African-born		Haitian-born		Jamaican-born		Other Caribbean-born		Other NHB immigrants	
	Years	APC	Years	APC	Years	APC	Years	APC	Years	APC	Years	APC	Years	APC
Male														
All sites combined	2005-2017	-4.57*	2005-2017	-4.76*	2005-2017	-5.23	2005-2017	-8.36	2005-2017	-3.82	2005-2017	-3.38	2005-2017	-13.79*
Prostate	2005-2017	-9.37*	2005-2017	-9.13*	2005-2017	-4.73	2005-2017	-15.16*	2005-2017	-5.48	2005-2017	-2.96	2005-2017	-24.27*
Lung and bronchus	2005-2017	-3.70*	2005-2017	-3.49	–	–	2005-2017	-12.03	2005-2017	-6.02	–	–	2005-2017	-3.38
Colon and rectum	2005-2017	-4.85*	2005-2017	-1.33	2005-2017	-4.50	2005-2017	-12.34	–	–	–	–	2005-2017	-5.42
Kidney and renal pelvis	2005-2015	-2.15*	2005-2017	-1.41	–	–	–	–	–	–	–	–	2005-2017	-11.40
	2015-2017	-26.22	–	–	–	–	–	–	–	–	–	–	–	–
Urinary bladder	2005-2015	-1.78	–	–	–	–	–	–	–	–	–	–	–	–
	2015-2017	-31.13	–	–	–	–	–	–	–	–	–	–	–	–
Female	Years	APC	Years	APC	Years	APC	Years	APC	Years	APC	Years	APC	Years	APC
All sites combined	2005-2012	-3.11*	2005-2017	-0.51	2005-2017	-1.86	2005-2017	-2.47	2005-2017	1.64	2005-2017	-0.36	2005-2017	1.55
	2012-2015	2.39	–	–	–	–	–	–	–	–	–	–	–	–
	2015-2017	-10.44*	–	–	–	–	–	–	–	–	–	–	–	–
Breast	2005-2017	-1.98*	2005-2017	-0.47	2005-2017	-1.18	2005-2017	4.78	2005-2017	3.86	2005-2017	-6.25	2005-2017	0.45
Lung and bronchus	2005-2015	-1.61*	2005-2017	-1.00	–	–	–	–	–	–	–	–	–	–
	2015-2017	-13.20	–	–	–	–	–	–	–	–	–	–	–	–
Colon and rectum	2005-2017	-5.35*	2005-2017	-1.18	2005-2017	-5.77	2005-2017	-4.76	2005-2017	4.38	–	–	2005-2017	1.29
Corpus and uterus NOS	2005-2017	0.77	2005-2017	2.53	–	–	–	–	2005-2017	-9.06*	–	–	2005-2017	4.06
Pancreas	2005-2017	0.99	2005-2017	0.60	–	–	–	–	–	–	–	–	–	–

^¤^Top 5 cancer sites based on NHB age-adjusted (6 age groups) invasive cancer incidence rates 2005-2017 by gender.

^1^Based on NAACCR NHIA, NJSCR Race 1, and Birthplace (state and county): NHIA = 0 (Non-Hispanic) and Race1 = 2 (Black).

^*^Annual percent change (APC) is significantly different from zero at alpha = 0.05.

-Unable to calculate APC due to there being zero case counts in some years.

Among NHB females, cancer incidence trends varied between 2005-2017. Among American-born females, cancer incidence for all sites combined decreased by 3.11% per year from 2005-2012 and 10.44% per year from 2015-2017, while a non-significant increase was recorded from 2012-2015. In this group, incidence of cancers of the breast (-1.98%), lung and bronchus (-1.61% for 2005-2015 only), and colon and rectum (-5.35%) significantly decreased. Although there were no significant trends for all sites combined or most site-specific cancers among NHB immigrant females overall or in subgroups, uterine cancer incidence among Jamaican-born females decreased significantly by 9.06%. Due to zero cases for some cancers among various NHB subgroups by birthplace, we could not calculate annual percent change for some site-specific cancers in some years.

## Discussion

This is one of few studies focusing on cancer incidence among NHB, disaggregated by American-born and immigrant subgroups. We observed evidence of within-group differences in age-adjusted cancer incidence rates by NHB subgroup in NJ. Notably, NHB immigrant males and females had lower cancer incidence rates than their American-born counterparts for all cancer sites combined and for several of the top five cancers diagnosed among NHBs. Cancer incidence was lower among Jamaican-born and other Caribbean-born females compared to African-born and Haitian-born females. Also, decreasing cancer incidence rates were largely significant for American-born NHBs, but less so for NHB immigrant subgroups. Prior studies examining the relationship between birthplace and cancer outcomes among Hispanic/Latinx populations have shown complex and inconsistent patterns, including a lower probability of being diagnosed with early-stage cancer (27) than their American-born counterparts, while also experiencing lower mortality rates (28). Neighborhood context, including ethnic density and poverty, and length of residence in the US, also influence cancer and other health outcomes differently across subgroups (29–31). More nuanced examination of the structural and neighborhood-level impacts on cancer outcomes is needed within NHB populations moving forward.

Lower cancer incidence rates were observed in the largest NHB immigrant groups compared to American-born NHBs for all cancer sites combined. This observation aligns with previous studies that report lower cancer mortality rates among NHB immigrant compared to American-born cases (20, 21, 32), suggesting higher cancer incidence and mortality rates among NHB compared to other racial groups is not only attributed to Black race. Explanations for lower cancer incidence among NHB immigrants may include the healthy immigrant effect and differences in lifestyle, attitudes, and perceptions among NHB subgroups. For example, tobacco exposure is the primary risk factor attributed to the development of many cancers. A recent study from the Cancer Prevention and Control Project of Philadelphia (CAP3) showed that Black immigrants have lower smoking prevalence compared to American-born Blacks (3.4% vs. 15%) (33). Findings also showed that as time in the US increased, immigrants had a 4% increase in the odds of ever smoking (33). Further research is, therefore, needed to better understand NHB subgroup differences in smoking behaviors to develop targeted interventions for tobacco exposure among NHB. Additionally, early interventions may be needed for NHB immigrants to prevent the increased likelihood of smoking as their time in the US increases.

The leading cancers among NHB males and females are prostate and breast cancer. We observed no difference in prostate cancer incidence between subgroups of immigrants and American-born NHB, suggesting similar biological risk factors (e.g., such as family history, genetics) and social determinants of health rooted in structural racism (34–37). Studies by the African Caribbean Cancer Consortium (AC3) have compared factors associated with prostate cancer risk between American-born and immigrant NHB men, showing that nativity did not significantly predict the likelihood of prostate cancer screening among NHB men (38). Other studies collectively support the role of genetic polymorphisms in the immune/inflammation genes associated with prostate cancer among both American-born and Caribbean-born NHB men (39–41). This observation is not unusual as there is mounting evidence that the immunologic/inflammatory pathways play an important role in prostate cancer biology among Black men in contrast to White men (42–44). Unlike prostate cancer, we observed lower breast cancer incidence among NHB immigrants compared to American-born females, which might indicate that breast cancer phenotypes differ across NHB subgroups. Among breast cancer cases diagnosed in South Florida from 2006-2017, Caribbean-born NHB immigrants were diagnosed with a larger proportion of estrogen receptor-positive (ER+) and progesterone receptor-positive (PR+) tumors compared to American-born NHB (ER+: 68.7% vs. 61%; *P* = 0.019 and PR+: 58.3% vs. 50.4%; *P* = 0.02) (45).

Lung and colorectal cancers are the second and third leading causes of cancer among NHB males and females. NHB immigrants have lower lung cancer incidence than their American-born counterparts. This is expected given the lower smoking prevalence among immigrant groups as described above (33). Kidney and bladder cancers are among the top five cancers for males, and for both cancers, NHB immigrants have lower incidence than American-born NHBs. Again, these differences might be attributed to differences in smoking behaviors between the two groups, as well as to differences in other relevant factors [e.g., diet and hypertension (46, 47)] across NHB groups. NHB immigrants also have lower colorectal cancer incidence than American-born NHBs. Recent findings from the CAP3 study showed that, while NHB immigrants are less likely to have health insurance, they are more likely to adhere to colorectal cancer screening than American-born NHBs (48). Therefore, differences in colorectal cancer incidence may not be related to access issues but to other factors, including diet (46) and neighborhood contextual factors (49, 50).

Uterine and pancreatic cancers are among the top five cancers among NHB females. NHB immigrants females have lower pancreatic cancer incidence than American-born NHBs, which may also be attributed to differences in smoking behavior. However, there was no significant difference in uterine cancer incidence between the two subgroups. This may be due to shared risk factors across birthplace subgroups (e.g., obesity, family history, and other lifestyle factors) (51). It is important to note that Caribbean immigrants are more often diagnosed with uterine cancer at a younger age and have worse survival than their American-born counterparts (51).

NHB immigrants were less likely than American-born NHBs to reside in census tracts with marked poverty, consistent with a national Pew Study showing that NHB immigrants aged >25 years were more likely to have a bachelor’s degree and less likely to live in a high-poverty neighborhood than American-born NHBs (52). Moreover, we found that NHB immigrant cases were more likely to be alive at the end of study follow-up. This could be attributed to the “immigrant paradox,” where recent immigrants report better overall health than their native-born peers or those who spent more time in the US because of differences in diet, acculturation, and other risk factors associated with cancer development (53). However, this has mostly been studied in Hispanic populations and requires further investigation in NHB populations (53). Differences in neighborhood contextual factors—which are rooted in structural racism (e.g., neighborhood disinvestment, food deserts, environmental chemical exposures)—might also contribute to differences in vital status (54–58).

An important limitation of this study was that missing birthplace data among NHB cancer cases in NJSCR records were relatively high (~38%) and could have led to underestimated cancer incidence rates. Deceased cancer cases are less likely to have unknown birthplace and nativity because death certificate is a major source for this information. This is supported by our findings of lower proportions of unknown birthplace for cancers that tend to be aggressive and/or have lower survival rates (e.g., lung and pancreas) and higher proportions of unknown birthplace for less aggressive cancers and/or those with higher survival rates (e.g., prostate and breast). Another interesting point is that that the proportion of NHB cancer cases with unknown birthplace is higher in recent years compared to earlier years. One reason for this might be that a larger proportion of cases diagnosed at the beginning of the study period (i.e., 2005-2009) are likely to be deceased compared to those diagnosed closer to the end of follow-up (i.e., 2015-2017). While some studies have reported variation in cancer mortality across NHB subgroups (20, 21, 32)—given greater availability of birthplace data among deceased cancer cases (59)—to our knowledge, to date, only one published study has reported variability in cancer incidence between some African-born and US-born individuals (18). Although information on birthplace is routinely collected in Surveillance, Epidemiology, and End Results (SEER) program registries, these data are missing for a large proportion of cases, likely in a non-random manner (60, 61). The percentage of missing data in our study is similar to cancer incidence studies that focused on Hispanic subgroups (up to 32%) (8, 62). To address missingness, prior studies have applied a series of approaches, including algorithms incorporating surname from cancer registries with (62) and without (8) linkage to death records. Most NHB cases in the current analysis with unknown birthplace were *in situ* or localized stage and <65 years at diagnosis, suggesting that combining incidence and death record data would not improve birthplace data missingness. As an alternative, studies in Hispanic subgroups have imputed missing birthplace using geographic location (62). This, combined with other data sources (e.g., birth records, death records), could further minimize missing birthplace data. Another consideration is that our simplistic definition of “NHB” race might have also led to an underestimation of cancer incidence rates. Relatedly, the use of ACS-based estimates to approximate NHB populations is subject to sampling errors. We also acknowledge that categorizing all African immigrants into one subgroup was not ideal given the geographically, culturally, and ethnically distinct populations that exist in Africa. However, insufficient case counts with birthplaces across multiple African countries (and geographic regions) limited our ability to further disaggregate the African-born subgroup. Nonetheless, we believe this study highlights some important differences in cancer incidence rates among NHB subgroups by birthplace and nativity—albeit in crudely disaggregated categories—that certainly warrant analysis in larger studies in the future. Lastly, the use of cancer registry data limited our ability to assess individual-level cancer risk factors that vary between NHB subgroups as explanations for the observed variation in cancer incidence. Despite the lack of complete data on birthplace and risk factor-related data, our findings add new knowledge about variation in cancer incidence, inclusive of Caribbean-born Black individuals in the US.

Despite these limitations, our novel data—generated from a population-based cancer registry in a state with substantial within-group variation in the NHB population—demonstrate differences in age-adjusted cancer incidence rates among NHBs by nativity and birthplace. Overall, cancer incidence for all sites combined and for the top five cancers, including some screen-detected cancers, was lower among NHB immigrants. Also, variation in cancer incidence trends by birthplace was observed. Improved collection of birthplace and African ancestry information in cancer registries is critically needed to enhance the ability to generate unbiased cancer surveillance statistics in disaggregated NHB groups by birthplace. These data are essential to understanding inequities and informing targeted strategies for cancer prevention and control, especially in subgroups shouldering a disproportionate burden.

## Data Availability Statement

The raw data supporting the conclusions of this article will be made available by the authors, without undue reservation.

## Ethics Statement

The studies involving human participants were reviewed and approved by Rutgers University Institutional Review Board. Written informed consent for participation was not required for this study in accordance with the national legislation and the institutional requirements.

## Author Contributions

Conceptualization: AL, JT, JG, AS. Data curation: JL, JG, KP. Formal analysis: JL, JG. Funding acquisition: AL, JT, AS. Methodology: AL, JT, JG, KP, AS. Writing – original draft: AL, FN, JT, SL, CR, AS. Writing – review & editing: All authors. All authors contributed to the article and approved the submitted version.

## Funding

This study was supported by the National Cancer Institute (Cancer Center Support Grant Number P30CA072720 provided funding support to AL and JT; 5P30CA006927 and 5U54CA221705 provided funding support to CR and SL; and R13CA249974 to the African Caribbean Cancer Consortium). New Jersey State Cancer Registry is funded by the National Cancer Institute’s Surveillance, Epidemiology and End Results (SEER) Program (#75N91021D00009), Centers for Disease Control and Prevention’s National Program of Cancer Registries (#5NU58DP006279) with additional support from the State of New Jersey and the Rutgers Cancer Institute of New Jersey. The funders had no role in the design of the study; the collection, analysis, and interpretation of the data; the writing of the manuscript; and the decision to submit the manuscript for publication.

## Conflict of Interest

The authors declare that the research was conducted in the absence of any commercial or financial relationships that could be construed as a potential conflict of interest.

## Publisher’s Note

All claims expressed in this article are solely those of the authors and do not necessarily represent those of their affiliated organizations, or those of the publisher, the editors and the reviewers. Any product that may be evaluated in this article, or claim that may be made by its manufacturer, is not guaranteed or endorsed by the publisher.
