# Supplementary material for: Variation in Cancer Incidence Rates Among Non-Hispanic Black Individuals Disaggregated by Nativity and Birthplace, 2005-2017: A Population-Based Cancer Registry Analysis
Source: Front Oncol. 2022 Apr 8;12:857548. doi: 10.3389/fonc.2022.857548 (PMC9024350; doi:10.3389/fonc.2022.857548)
Supplement: Supplementary file 1 [file Table_1.docx]

| **Supplementary Table 1.** Proportion of unknown birthplace and nativity among NHB invasive cancer cases diagnosed in New Jersey between 2005-2017 by year of diagnosis | |
| --- | --- |
| **Year of diagnosis** | **Proportion of cases with unknown birthplace and nativity** |
| 2005 | 28.6% |
| 2006 | 31.1% |
| 2007 | 31.9% |
| 2008 | 32.8% |
| 2009 | 34.8% |
| 2010 | 37.9% |
| 2011 | 38.1% |
| 2012 | 39.1% |
| 2013 | 38.8% |
| 2014 | 37.8% |
| 2015 | 40.9% |
| 2016 | 44.6% |
| 2017 | 50.2% |
| Mean±SD | 37.7±0.1% |
