# Supplementary material for: Variation in Cancer Incidence Rates Among Non-Hispanic Black Individuals Disaggregated by Nativity and Birthplace, 2005-2017: A Population-Based Cancer Registry Analysis
Source: Front Oncol. 2022 Apr 8;12:857548. doi: 10.3389/fonc.2022.857548 (PMC9024350; doi:10.3389/fonc.2022.857548)
Supplement: Supplementary file 2 [file Table_2.docx]

| **Supplementary Table 2.** Counts and proportions of NHB invasive cancer cases diagnosed in New Jersey between 2005-2017, by year of last contact, birthplace and nativity, and vital status | | | | | | |
| --- | --- | --- | --- | --- | --- | --- |
|  | **American-born** | | | **NHB Immigrants** | | |
| **Year of Last Contact** | **Total**  **n (%)** | **Deceased**  **n (%)** | **Alive**  **n (%)** | **Total**  **n (%)** | **Deceased**  **n (%)** | **Alive**  **n (%)** |
| 2005 | 796 (2.0) | 796 (2.6) | 0 (0.0) | 82 (1.5) | 77 (2.4) | 5 (0.2) |
| 2006 | 1,402 (3.6) | 1,397 (4.5) | 5 (0.1) | 71 (1.3) | 57 (1.8) | 14 (0.6) |
| 2007 | 1,678 (4.3) | 1,673 (5.4) | 5 (0.1) | 104 (1.9) | 86 (2.7) | 18 (0.8) |
| 2008 | 1,975 (5.1) | 1,966 (6.3) | 9 (0.1) | 169 (3.1) | 140 (4.4) | 29 (1.3) |
| 2009 | 2,073 (5.3) | 2,064 (6.6) | 9 (0.1) | 221 (4.1) | 191 (5.9) | 30 (1.4) |
| 2010 | 2,196 (5.7) | 2,183 (7.0) | 13 (0.2) | 246 (4.5) | 224 (7.0) | 22 (1.0) |
| 2011 | 2,312 (6.0) | 2,292 (7.4) | 20 (0.3) | 243 (4.5) | 207 (6.4) | 36 (1.6) |
| 2012 | 2,306 (5.9) | 2,286 (7.4) | 20 (0.3) | 286 (5.3) | 240 (7.5) | 46 (2.1) |
| 2013 | 2,447 (6.3) | 2,427 (7.8) | 20 (0.3) | 321 (5.9) | 285 (8.9) | 36 (1.6) |
| 2014 | 2,585 (6.7) | 2,558 (8.2) | 27 (0.3) | 316 (5.8) | 276 (8.6) | 40 (1.8) |
| 2015 | 2,738 (7.1) | 2,706 (8.7) | 32 (0.4) | 332 (6.1) | 294 (9.1) | 38 (1.7) |
| 2016 | 2,789 (7.2) | 2,743 (8.8) | 46 (0.6) | 411 (7.6) | 362 (11.2) | 49 (2.2) |
| 2017 | 2,945 (7.6) | 2,863 (9.2) | 82 (1.1) | 437 (8.0) | 379 (11.8) | 58 (2.6) |
| 2018 | 2,267 (5.8) | 2,039 (6.6) | 228 (2.9) | 394 (7.3) | 274 (8.5) | 120 (5.4) |
| 2019 | 8,325 (21.4) | 1,086 (3.5) | 7,239 (93.3) | 1,800 (33.1) | 126 (3.9) | 1,674 (75.6) |
| Total | 38,834 (100.0) | 31,079 (100.0) | 7,755 (100.0) | 5,433 (100.0) | 3,218 (100.0) | 2,215 (100.0) |
